# The ElonginB/C-Cullin5-SOCS-Box-Complex Is a Potential Biomarker for Growth Hormone Disorders

**DOI:** 10.3390/biomedicines9020201

**Published:** 2021-02-17

**Authors:** Wilhelm Gossing, Lars Radke, Henrik Biering, Sven Diederich, Knut Mai, Marcus Frohme

**Affiliations:** 1 Division Molecular Biotechnology and Functional Genomics, Technical University of Applied Sciences, 15745 Wildau, Germany; wilhelm.gossing@th-wildau.de (W.G.); lradke@th-wildau.de (L.R.); biering@th-wildau.de (H.B.); 2 Department of Endocrinology and Metabolism, Charité—Universitätsmedizin Berlin, Corporate Member of Freie Universität Berlin, Humboldt-Universität zu Berlin, and Berlin Institute of Health, 10117 Berlin, Germany; Sven.Diederich@medicover.de (S.D.); knut.mai@charite.de (K.M.); 3 Praxis an der Kaisereiche, Innere Medizin, Endokrinologie, Diabetologie, 12159 Berlin, Germany; 4 MVZ Medicover Berlin-Mitte, Innere Medizin, Endokrinologie, Andrologie, 10117 Berlin, Germany; 5 German Centre for Cardiovascular Research (DZHK), Partner Site Berlin, 10117 Berlin, Germany

**Keywords:** acromegaly, growth hormone deficiency, biomarker, SOCS2, liquid biopsies, pituitary

## Abstract

Insulin-like growth factor 1 (IGF-1) is the standard biochemical marker for the diagnosis and treatment control of acromegaly and growth hormone deficiency (GHD). However, its limitations necessitate the screening for new specific and sensitive biomarkers. The elonginB/C-cullin5-SOCS-box-complex (ECS-complex) (an intracellular five-protein complex) is stimulated by circulating growth hormone (GH) and regulates GH receptor levels through a negative feedback loop. It mediates the cells’ sensitivity for GH and therefore, represents a potent new biomarker for those diseases. In this study, individual ECS-complex proteins were measured in whole blood samples of patients with acromegaly (n = 32) or GHD (n = 12) via ELISA and compared to controls. Hierarchical clustering of the results revealed that by combining the three ECS-complex proteins suppressor of cytokine signaling 2 (SOCS2), cullin-5 and ring-box protein 2 (Rbx-2), 93% of patient samples could be separated from controls, despite many patients having a normal IGF-1 or not receiving medical treatment. SOCS2 showed the best individual diagnostic performance with an overall accuracy of 0.93, while the combination of the three proteins correctly identified all patients and controls. This resulted in perfect sensitivity and specificity for all patient groups, which demonstrates potential benefits of the ECS-complex proteins as clinical biomarkers for the diagnostics of GH-related diseases and substantiates their important role in GH metabolism.

## 1. Introduction

Growth hormone deficiency (GHD) and acromegaly are two growth hormone disorders caused by reduced or increased levels of growth hormone (GH), respectively. Those diseases are mainly triggered by pituitary neuroendocrine tumors (PitNETs) that slowly shift the physiological balance of hormone secretion to pathological levels. While PitNETs appear to be rather common within the general population, with 17%, only a very small percentage (<0.04%) of affected people develops symptoms of a GH disorder that requires medical attention [1,2]. Regardless, the prevalence of acromegaly is approximately 60–100 cases per million and seems to be on the rise, probably owing to the increased awareness and better data availability amongst physicians [3,4,5,6,7]. On the other hand, a GHD is often caused by a non-functioning PitNET and is reported to occur in 290–460 cases per million, thus being more frequent than acromegaly [8].

Because symptoms of GH-related disorders evolve very subtly over the course of many years, initial diagnoses are made only after 5–25 years [9]. This results in 73% of cases already being macroadenomas (>1 cm) upon diagnosis, which significantly reduces the surgical cure rate [10,11]. While surgical removal of microadenomas is effective in approximately 80% of cases, it is successful in 55% of macroadenomas only [12]. If residual tumor activity is observed, medical control of GH and insulin-like growth factor 1 (IGF-1) secretion is usually required. Moreover, GHD and acromegaly both involve a high morbidity and increased mortality that often persist even after surgical intervention [13]. The decreased life expectancy is mainly caused by cardiovascular and respiratory diseases [14]. Especially suboptimal disease control is associated with poor outcome. Given these data, early diagnosis as well as accurate evaluation of disease activity is highly required.

For the diagnosis of GH disorders, it is suggested to gather biochemical evidence by performing at least one laboratory test [15]. Such provocative tests are time-consuming and include complex and partially hazardous procedures [16]. Additionally, they are considered unreliable, variable and do lack reproducibility [17]. Therefore, IGF-1 is used to complement the diagnostics but also to monitor treatment. However, IGF-1 is said to be not useful for screening and can give conflicting results, as its regulation is dependent on factors such as age, gender or nutritional status [18,19,20]. For patients with a GHD this is especially problematic, as there are considerable overlaps with the normal range that further increase with age [21]. Of major concern is the lack of standardization of IGF-1 assays and the variability of applied clinical reference ranges. Laboratories use different methods for IGF-1 detection, which results in the limits of applied reference ranges varying by more than twofold [22,23]. For the screening of a GHD, the additional measurement of insulin-like growth factor binding protein-3 (IGFBP-3) can be useful. Its use, however, is equally controversial. While some studies showed that due to its low sensitivity the use of IGFBP-3 does not provide an advantage over the measurement of IGF-1 alone [24,25], others highlight the significantly higher specificity of IGFBP-3 [18,26].

The response to medical treatment is highly individual and neither provocative tests nor biochemical markers are able to reliably predict treatment control [27]. This may in part be due to effects of GH that are not mediated via its hepatic metabolism, meaning that IGF-1 is not representing the physiological efficacy of GH [28].

Several potential alternatives to IGF-1 have been proposed but despite clinical correlation with GH-related diseases in those markers, none of them got established in clinical diagnostics [29,30,31,32,33]. So there is still the need for a more accurate biomarker, which can be used for diagnosis as well as assessment of disease activity [34]. Furthermore, physicians also suggest a re-evaluation of the biomarkers in use, due to new available treatment options, such as long-acting drugs [35].

Here, we investigated a novel set of potential biomarkers that have a close connection to the physiological action of GH. The molecule of interest is the elonginB/C-cullin5-SOCS-box-complex (ECS-complex) which consists of five proteins: Suppressor of cytokine signaling 2 (SOCS2), elongin B (EloB), elongin C, cullin-5 and ring-box protein 2 (Rbx-2). This complex controls GH signaling by regulating the GH receptor (GHR) through a negative feedback loop. As the GH uptake of target cells is primarily dependent on the GHR expression, the proteins of the ECS-complex are assumed to reflect the individual cellular response characteristics towards GH [36]. The protein SOCS2 is the part of the ECS-complex that recognizes and binds to the GHR. More precisely, SOCS2′s SH2-domain associates to the phosphorylated tyrosines Y487 and Y595 of the GHR [37,38]. Mutations to these two amino acids lead to a complete loss of function of the ECS-complex [39]. GH is a direct stimulus for the expression of SOCS2. Its induction is slow and steady over the course of 24 h, which suggests independence of daily GH fluctuations [40]. This would be advantageous for clinical applications as the time of sampling was arbitrary. A connection between SOCS2 and acromegaly has already been proven, when significant increases in SOCS2 expression were detected in adipose tissue of acromegalic patients [41]. However, the inhibitory action of high SOCS2 levels apparently did not stop the development of acromegaly. At its C-terminal end, SOCS2 holds a SOCS-box domain which allows the association to the proteins elongin B, elongin C and cullin-5 [42]. The 91 kDa protein cullin-5 acts as a scaffold to bring the substrate recognition unit SOCS2 into close proximity to the ubiquitin-ligase Rbx-2. The elongins B and C are important for the correct folding and stabilization of SOCS2. Rbx-2 is an E3 ligase that attaches to cullin-5. It carries a RING-motif that mediates the transfer of ubiquitin from an E2 ubiquitin-conjugating enzyme to the GHR [43].

**Figure 1:**
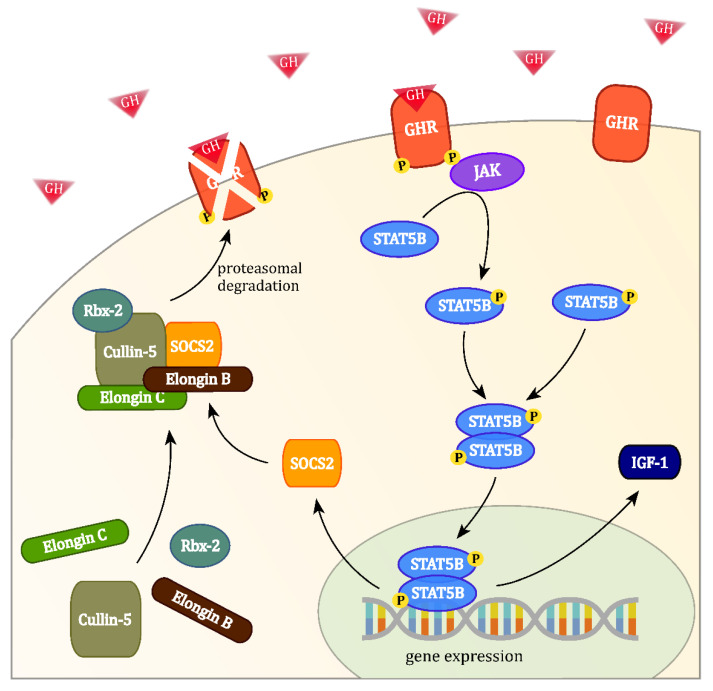
The binding of growth hormone (GH) at the GH receptor (GHR) activates the intracellular Janus Kinase/Signal Transducer and Activator of Transcription signalling cascade, which induces the expression of suppressor of cytokine signaling 2 (SOCS2) and leads to the formation of the elonginB/C-cullin5-SOCS-box-complex (ECS-complex). Phosphorylated GHRs may be targeted by the ECS-complex for ubiquitin transfer, leading to their proteasomal degradation.

The regulatory action of the ECS-complex takes place, when GH binds to the GHR. This phosphorylates the receptor and activates the intracellular Janus Kinase/Signal Transducer and Activator of Transcription pathway, which induces the expression of SOCS2. Together with the other four proteins, SOCS2 subsequently forms the ECS-complex in the cytoplasm and is then able to bind to phosphorylated tyrosines at the GHR. This allows Rbx-2 to transfer ubiquitin moieties to the GHR, which in turn is recognized and degraded by the proteasome (see Figure 1). SOCS2 does not bind to unphosphorylated receptors, possibly keeping the cell sensitive to low levels of GH in the blood [39].

The GHR shows differential expression in peripheral blood mononuclear cells (PBMCs), which suggests that the regulatory ECS-complex is active in these cells [44]. A GH-dependent regulation of the ECS-complex gene expression in patients with acromegaly and GHD was also already demonstrated [45]. In this study, we investigated the protein expression of the ECS-complex in blood samples of patients with diagnosed GH disorders and healthy controls for their benefits in personalized medicine.

## 2. Materials and Methods

### 2.1. Study Subjects

A positive approval from the Ethics Committee of the Landesärztekammer Berlin (11/03/2013, ID number: THW-GHD-2012) was obtained previous to starting the study and all participants gave their written informed consent before blood withdrawal. The control group participants were recruited in a local general practitioner’s surgery. They had to be above the age of 40 and were without known neoplasms or hormonal disorders. Common cold or flu were exclusion criteria, and normal IGF-1 values were verified to eliminate possible GH-related abnormalities in the control group. For the experimental groups, we included patients from four endocrine surgical departments who had been diagnosed with either acromegaly or GHD. The diagnosis of acromegaly was determined by IGF-1 and inadequate suppression of serum GH to <0.4 ng/mL by oral glucose load. GHD was diagnosed by IGF-1 and insufficient GH response to insulin-induced hypoglycemia.

### 2.2. Sample Preparation

From every subject, a single whole-blood sample of 5 mL was obtained during their routine medical consultation. Blood samples were drawn between 7 and 12 am, were stored in K_2_/EDTA vacutainers, put immediately on ice and were transported to the laboratory within a few hours. Red blood cells were lysed with 0.17 M ammonium chloride and PBMCs collected from 2 mL of whole blood by two centrifugation cycles of 5 min at 453× *g*. After washing, pelleted PBMCs were frozen at −80 °C.

### 2.3. Assays

IGF-1 measurements of serum from patients and controls were conducted with the Immulite 2000 (Siemens AG). For the classification of the samples, the age-dependent in-house reference range was used (Praxis an der Kaisereiche, 12159 Berlin, Germany).

For the protein measurements, PBMCs extracted from whole blood samples were thawed in cold phosphate buffered saline (PBS) and the cell concentration was determined with an automated cell counting system (Cedex XS, Roche Innovatis AG). Afterwards, cells were diluted to 4 × 10^6^ cells per mL in PBS and homogenized using the Precellys (Bertin Instruments) and 1.4 mm zirconium oxide beads at 5.000 rpm for 15 s. The beads were removed, and the solution was centrifuged at 5.000× *g* for 5 min. The resulting supernatant contained the proteins of interest.

The levels of the ECS-complex proteins SOCS2, EloB, cullin-5 and Rbx-2 were measured with sandwich enzyme-linked immunosorbent assay kits purchased from Abbexa Ltd. (Cambridge, UK) in accordance with the manufacturer’s instructions. Due to a lack of an appropriate ELISA kit or antibody, the ECS-complex component elongin C was not analyzed in this study.

### 2.4. Statistics

All data are given as mean ± standard deviation (SD) unless stated otherwise. Pearson correlation coefficients and unpaired t-tests were calculated using GraphPad Prism 5. Graphics were created with R statistics v3.5.2. P-values lower than 0.05 were considered significant. Protein concentrations have been estimated from the signal intensities of ELISA tests with the five parameter logistic regression model (5PL) of Prentice [46] and normalized to one million cells. Outlier detection of control group values was done using the normalized median absolute deviation as proposed by Farazi and Imon [47]. Clustering was performed using Ward’s hierarchical agglomerative clustering method [48]. Protein combinations have been obtained for each of the four subgroups individually by averaging weighted standard deviation scores $x_{i}$ based on their accuracy in the respective subgroup (Equation (1)) [49].

(1) $$x_{i}=\log\left(\frac{accuracy_{i}}{1-accuracy_{i}}\right),\;i\;\in \;\{\mathrm{SOCS}2,\;\mathrm{Cullin}-5,\;\mathrm{Rbx}-2\}$$

## 3. Results

### 3.1. Patient Characteristics

The control group cohort (*n* = 14, male = 8, female = 6) had a mean age of 62.9 ± 13.1 years and a mean body-mass-index (BMI) of 27.1 ± 4.8 kg/m^2^ (see Table S1). The group of patients included 32 samples from patients with a diagnosed acromegaly (age = 48.2 ± 14.1, BMI = 29.8 ± 5.7 kg/m^2^) and 12 samples from patients with a diagnosed GHD (age = 51.0 ± 11.7, BMI = 28.0 ± 4.5 kg/m^2^). The patients were separated by their type of GH-related disorder (acromegaly/GHD) and further divided into preoperative and postoperative, respectively. Nine newly diagnosed patients with acromegaly and five with a newly diagnosed GHD, who had not received any treatment yet, were assigned to the preoperative subgroups. The patients that donated blood to the study after their tumor surgery were assigned to the postoperative subgroups. The postoperative acromegaly group (n = 23) contained 15 samples with GH suppressive medication and eight samples that did not receive any medication. For postoperative GHD (*n* = 7), four samples were from patients who received daily GH replacement therapy while three did not receive treatment. All postoperative patients had a normal IGF-1 and therefore a controlled disease. A significant difference in mean age between all patients and controls existed (*p* = 0.002), but mean BMI values did not differ significantly (*p* = 0.08).

### 3.2. IGF-1

**Figure 2:**
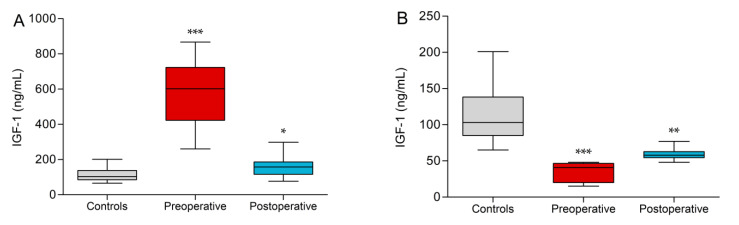
Insulin-like growth factor 1 (IGF-1) values of acromegaly subgroups (**A**) and growth hormone deficiency (GHD) subgroups (**B**) vs. controls. Significant differences to controls are indicated (* *p* < 0.05, ** *p* < 0.01, *** *p* < 0.001).

The control group had a mean IGF-1 of 113.8 ± 39.4 ng/mL and all controls were within the age-dependent clinical reference range (RR). All preoperative acromegaly patients had IGF-1 levels above the clinical RR. The values were highly elevated in comparison to the control group (580.9 ± 202.6 ng/mL, *p* < 0.0001). Although their samples were within the clinical RR, the postoperative acromegaly group showed a significant increase in IGF-1 values vs. controls (155.2 ± 51.4 ng/mL, *p* = 0.01, Figure 2A). The GHD patient groups showed significant reductions of IGF-1 vs. controls (34.8 ± 14.2 ng/mL, *p* = 0.0005) but only the five preoperative GHD patients fell below the clinical RR. Postoperative GHD patients showed normal values, which were close to the lower reference range boundary but still lower than in controls (58.8 ± 8.8 ng/mL, *p* = 0.002, Figure 2B).

### 3.3. ECS-Complex

**Figure 3:**
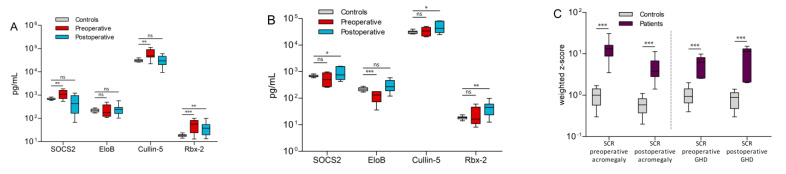
Comparison of ECS-complex protein concentrations in controls vs. acromegaly (**A**) and GHD patients (**B**). A protein index named SCR was calculated by combining the weighted z-scores of SOCS2, cullin-5 and Rbx-2. This SCR value significantly improved separation between patients and controls in all acromegaly and GHD subgroups (**C**). Significance levels are indicated above the horizontal lines (ns = not significant, * *p* < 0.05, ** *p* < 0.01, *** *p* < 0.001).

The results of the ELISA tests for the control group samples showed protein concentrations of 690.9 ± 68.7 pg/mL for SOCS2, 223.3 ± 36.0 pg/mL for EloB, 31.1 ± 3.9 ng/mL for cullin-5 and 18.5 ± 2.4 pg/mL for Rbx-2. Conversely, the values from the experimental groups were much more dispersed. Preoperative acromegaly patients were characterized by higher levels of SOCS2 (1128.8 ± 471.0 pg/mL, *p* = 0.003), cullin-5 (60.99 ± 30.29 ng/mL, *p* = 0.001) and Rbx-2 (56.4 ± 31.1 pg/mL, *p* = 0.0002) compared to controls. Only EloB showed no significant difference to controls (243.8 ± 153.4 pg/mL, *p* = n.s.). In contrast, most of these differences were not observed comparing the postoperative acromegaly group and controls (Figure 3A). Only in Rbx-2, a significant difference to controls persisted (*p* = 0.001). Interestingly, the highest measured values of the four proteins derive from a patient, who was with 260 ng/mL IGF-1 barely above the clinical RR. In preoperative GHD patients, only EloB was lower than in controls (126.8 ± 58.8 pg/mL, *p* = 0.0004, Figure 3B). In comparison, higher levels of SOCS2 (*p* = 0.04), cullin-5 (*p* = 0.01) and Rbx-2 (*p* = 0.001) could be found in postoperative GHD samples compared to control samples. Although there was a significant difference in mean age between patients and controls, there was no linear dependency on age in SOCS2 (*r* = 0.04, *p* = 0.89), EloB (*r* = 0.29, *p* = 0.32), cullin-5 (*r* = −0.5, *p* = 0.07) or Rbx-2 (*r* = −0.09, *p* = 0.75). Moreover, BMI values, which ranged from 18 to 35 kg/m^2^ in controls, also did not correlate with measured SOCS2 (*r* = 0.24, *p* = 0.43), EloB (*r* = 0.09, *p* = 0.76), cullin-5 (*r* = −0.03, *p* = 0.91) or Rbx-2 (*r* = 0.14, *p* = 0.64) levels. Furthermore, no significant gender differences in mean SOCS2 (*p* = 0.54), EloB (*p* = 0.13), cullin-5 (*p* = 0.43) or Rbx-2 (*p* = 0.35) protein concentrations were observed in controls. By clustering the results of all samples, the scaled combination of SOCS2, cullin-5 and Rbx-2 formed three clusters with all control samples falling into one cluster (Figure S1, green box). That way, 42 out of 44 patients (93%) could be distinguished from controls. However, clustering was not useful to discriminate between acromegaly and GHD or between preoperative and postoperative. Due to these clustering results, the three proteins SOCS2, Cullin-5 and Rbx-2 were combined into a single value for each subgroup separately, which we named SCR. This value improved the separation between every patient subgroup and the control group significantly (*p*-values < 0.0001, Figure 3C).

Next, diagnostic performance characteristics of the ECS-complex proteins were calculated for all four subgroups individually. All patients were assessed as positive, regardless of their treatment status (controlled or uncontrolled). Receiver operating characteristic (ROC) curves have been compiled to calculate area under the curve (AUC) values and to investigate the trade-off between sensitivity (SEN) and specificity (SPEC).

**Figure 4:**
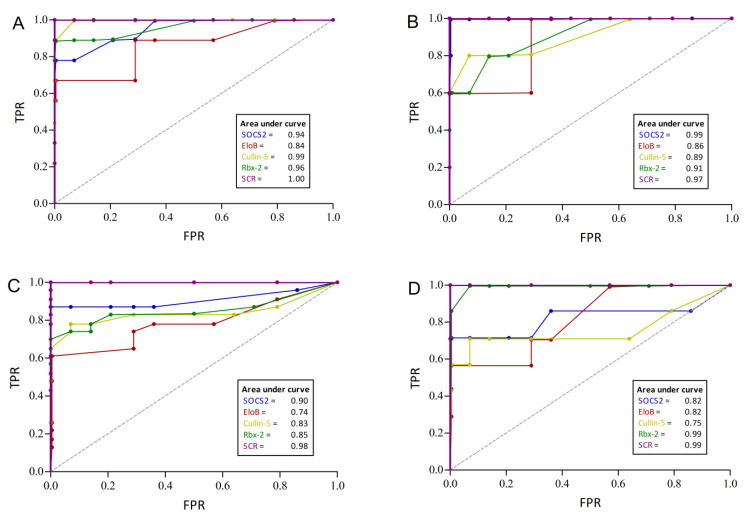
Performance characteristics of ECS-complex proteins to discriminate patients with acromegaly or GHD from controls. The receiver operating characteristic (ROC) curves depict the diagnostic power for preoperative acromegaly (**A**), preoperative GHD (**B**), postoperative acromegaly (**C**) and postoperative GHD (**D**), respectively. The purple graph named SCR combines SOCS2, cullin-5 and Rbx-2 and shows the best performance in all four patient groups. Area under the curve values are presented in the small boxes. TPR = true positive rate; FPR = false positive rate.

For preoperative acromegaly (Figure 4A), SOCS2, cullin-5 and Rbx-2 showed high diagnostic accuracies with 0.91, 0.96 and 0.96, respectively. The highest SENs were achieved by cullin-5 and Rbx-2 with 1.00 and 0.89, respectively. The SEN for detecting preoperative GHD patients ranged from 0.60 (EloB and Rbx-2) to 1.0 in SOCS2. SOCS2 also correctly rejected all controls for a specificity of 1.0 and the resulting AUC was 0.99 (Figure 4B). In comparison, the ECS-complex proteins showed lower SENs in the postoperative groups, probably owing to lower disease activities in these patients. Still, SOCS2 showed a good SEN and SPEC in postoperative acromegaly (0.87 and 1.0), that translated to the highest AUC in that subgroup (0.90, Figure 4C). Similarly, Rbx-2 identified the most postoperative GHD patients correctly while testing negative for all controls (SEN = 0.86, SPEC = 1.0). This protein showed a high discriminative power with an AUC of 0.99 (Figure 4D).

In all four groups, the combined SCR index correctly classified all patients and controls in all tests. Furthermore, its SEN and SPEC was always 1.00 and the AUCs of the SCR index further substantiate its excellent diagnostic accuracy, as they range from 0.97 to 1.00 (see Figure 4A–D).

## 4. Discussion

This study is the first to investigate changes of all ECS-complex proteins in blood samples of patients with treatment-naïve or controlled GH disorders. Our data provide evidence for a correlation of these disorders with abnormal concentrations of ECS-complex proteins. While protein concentrations were significantly elevated in preoperative acromegaly, they were found to occur both elevated or reduced in GHD. The reasons for these discrepancies are not understood yet, as they could not be attributed to certain characteristics or therapy states of individual patients.

In order to conform to the typical age distribution of patients with GH-related disorders, only controls above 40 years of age were recruited for the study. This resulted in a significantly higher mean age in controls vs. patients but did not seem problematic as none of the proteins indicated an age-related correlation. Problematic, however, is the broad reference range of the standard biomarker IGF-1, because it may complicate reliable diagnostic assessments as well as treatment control. In contrast, the ECS-complex proteins show a stable physiological expression due to small coefficients of variation which suggests narrow reference ranges. This would allow more precise diagnostics and furthermore may explain the measured abnormal ECS-complex concentrations in postoperative patients with normalized IGF-1 levels. Accordingly, these values could indicate that many patients may still harbor residual disease activity. This assumption is supported by research showing that the IGF-1 of patients with acromegaly can normalize with symptoms still in place [50]. Similarly, IGF-1 values of symptomatic GHD patients often overlap with the reference range [21]. Therefore, the narrower reference ranges of the ECS-complex proteins could enable the detection of such mild activity and thus may help to optimize treatment efficacy.

Furthermore, no correlations between age, gender or BMI and the ECS-complex proteins in the control group samples have been found. Therefore, these biometric data may not have to be considered when using these proteins as biomarkers. It may also mean that the known gender-related effects of estrogen on the GH/IGF-1 axis may not affect the ECS-complex [51]. This also challenges the reported increases of SOCS2 expression after estrogen treatment in mice, which were achieved with superphysiological concentrations of estradiol, that do not occur naturally in humans [52]. Regardless, the results about the effects of age, gender and BMI on the ECS-complex may be limited due to the rather small sample size. Therefore, a confirmation in a larger cohort is necessary. The significant elevations of SOCS2, cullin-5 and Rbx-2 in the preoperative acromegaly group show that these proteins might be candidates for disease detection. The changes of all ECS-complex proteins in postoperative acromegaly patients and of SOCS2 and EloB in postoperative GHD patients are smaller in comparison to the preoperative groups, which indicates a detectable attenuation of disease severity after surgery. Interestingly, the group separation could be enhanced much further through combination of SOCS2, cullin-5 and Rbx-2, which clearly shows that a joint measurement of the three proteins would be much more efficient than just a single marker. The results of individual patients reveal correlations between the ECS-complex proteins which suggests that they have a common underlying regulation mechanism. The sole exception to this is Rbx-2, which is often increased when the other proteins are not. It therefore appears to be regulated independently. Our results support the findings of Hochberg et al., who discovered that SOCS2 mRNA expression is increased only in patients with acromegaly below 60 years [41]. In our study, the median of SOCS2 in acromegaly patients over 60 years was increased by 15% compared to controls, while it was increased by 57% in patients below 60 years (*p* = 0.008). As this age-dependent effect could not be observed in controls, it may only apply to the stimulation of SOCS2 without affecting its basal levels. It further indicates that the SOCS2-dependent feedback loop is more active in younger patients, while it weakens in the elderly. Ultimately, this could impede the diagnostic capability of SOCS2 in older patients. In mice, SOCS2 was found to only carry out its inhibitory effect on GH signalling in lower concentrations, while it potentiated signalling at higher concentrations [53,54]. Accordingly, the predominantly elevated concentrations of SOCS2 in the preoperative acromegaly patients are apparently not sufficient to fulfill its biological protective function against the elevated levels of circulating GH. However, SOCS2 is downregulated in 60% of preoperative GHD patients, where an inhibition of the GHR by the ECS-complex is not desirable. Therefore, the question arises, what concentrations would be considered “high” or “low” for the human body, respectively, for SOCS2 to have an inhibiting or potentiating effect.

The statistical capacity of the ECS-complex proteins as diagnostic biomarkers was estimated by comparing their performance parameters. Generally, the SPEC of all proteins was 100% in nearly all tests, which is probably due to the low variation of measured ECS-complex protein concentrations in controls. Only for cullin-5 in the preoperative groups, the calculated SPEC was lower (93%).

Of the individual proteins, SOCS2 showed the best performance on average, as its diagnostic accuracy was over 0.9 in all four groups. Its advantage over the other ECS-complex proteins might be its direct stimulation by GH. Therefore, it may be of good use as a single biomarker for both acromegaly and GHD. However, clustering of the results revealed that a combination of SOCS2, cullin-5 and Rbx-2 yielded a better separation of patients and controls than any of the individual proteins. This accounted for all four subgroups including preoperative GHD, where EloB showed the highest significance but worsened the group separation. In fact, by using the SCR index, the SEN and overall diagnostic performance was further improved. The SCR index was able to correctly identify every patient as positive and every control as negative, therefore presenting perfect SEN and SPEC in all four groups. It also identified patients with a normal IGF-1 as positive, while rejecting all controls. On the contrary, studies assessing the value of IGF-1’s diagnostic capabilities for these diseases are controversial. A meta-analysis determined IGF-1’s SEN (0.66), SPEC (0.69) and AUC (0.78) for the diagnosis of a GHD, which indicates only moderate performance [18]. Because of the indistinct demarcation in the low concentration range, the diagnosis of a GHD with IGF-1 is the most unreliable. Having an alternative for these cases could therefore be very valuable. Additionally, the many observed differences of ECS-complex proteins in postoperative patients with a normal IGF-1 suggest that these patients cannot be classified as controlled. Therefore, our results speak for a superior detection of pathological conditions in postoperative patients or during treatment using the SCR index. Unfortunately, comparable statistics of IGF-1 for acromegaly have not been found, but our results suggest an excellent diagnostic performance using the three ECS-complex proteins SOCS2, cullin-5 and Rbx-2 in conjunction.

Lastly, there are some limitations in our study that should be pointed out. The comparison of the here investigated biomarkers to IGF-1 is biased because it was part of the initial diagnostic assessment for the patients in this study. Nonetheless, it can be added that the absence of correlation between any of the ECS-complex proteins and IGF-1 was expected as SOCS2-deficient mice do not show elevated IGF-1 expression after GH treatment [55]. For a better comparison of the diagnostic capabilities of the ECS-complex to IGF-1, a quality of life (QoL) questionnaire would have been helpful, as it measures the patients’ perception of disease control. The assessment of QoL was shown to be able to detect relevant clinical improvements without a significant change in IGF-1 [56]. Moreover, consecutive data on ECS-complex concentrations from single patients during medical dose optimization have not been obtained and might have been helpful for determining its capabilities for treatment monitoring and control. Further limiting was our control group size of 14 healthy volunteers, which were the only source for reference data and for the establishment of a normal range of ECS-complex concentrations. These controls were not strictly matched to the biometric data of recruited patients, so that the difference in age could have possibly played a part in the observed changes. Future studies should verify our findings with a larger set of patients to learn more about the role of the ECS-complex in the GH metabolism and to possibly leverage its significance as a new biomarker.

## 5. Conclusions

This is the first study to evaluate the potential of the ECS-complex for the diagnostics of GH-related diseases. We have demonstrated significant differences in all four investigated ECS-complex proteins between patients with acromegaly or GHD and healthy controls. The stable expression of the proteins in controls together with the significant differences in patients make up good characteristics for them to be used as alternative biomarkers. Measured protein concentrations also did not change in relation to age, gender or BMI, indicating independence from these factors.

Overall, SOCS2 showed the best clinical performance in terms of SEN, SPEC and accuracy as an individual marker. However, superior results were obtained with a combination of SOCS2, cullin-5 and Rbx-2, which showed excellent diagnostic performance by reliably separating all patients from controls.

Therefore, this combination of proteins is proposed as a novel biomarker candidate for acromegaly as well as GHD and because of its high sensitivity can be considered for the screening of such rare diseases as well. In the future, it may be an alternative for contradictory IGF-1 results, postoperative treatment control or hazardous provocative tests.

## Data Availability

The data presented in this study are available on request from the authors. The data are not publicly available due to privacy reasons.

## Supplementary Materials

The following are available online at https://www.mdpi.com/2227-9059/9/2/201/s1, Table S1: Individual biometric and protein data of control group participants., Figure S1: Cluster analysis of SOCS2, cullin-5 and Rbx-2 values in all study participants.
